# A preliminary effect analysis of family doctor and medical insurance payment coordination reform in Changning District of Shanghai, China

**DOI:** 10.1186/s12875-019-0949-0

**Published:** 2019-05-10

**Authors:** Jiaoling Huang, Wei Lu, Luan Wang, Tao Zhang, Chengjun Liu, Shanshan Liu, Hong Liang, Yimin Zhang, Dongfeng Guo

**Affiliations:** 1 School of Public Health, Shanghai Jiao Tong University School of Medicine, Shanghai, China; 2 School of Economics and Management, Hainan Normal University, Hainan, China; 3 Shanghai Sixth People’s Hospital East Affiliated to Shanghai University of Medicine &Health Sciences, Shanghai, China; 4 Jinyang Community Health Service Center of Pudong New Area, Shanghai, China; 5 School of Social Development and Public Policy, Fudan University, Shanghai, China; 6 Pudong Institute for Health Development, Shanghai, China; 7 Eye and Dental Diseases Prevention & Treatment of Pudong New Area, Shanghai, China; 8 Department of General Medicine, Gongli Hospital, The Second Military Medical University, Shanghai, China

## Abstract

**Background:**

Changning District of Shanghai pioneered in implementing Family Doctor and Medical Insurance Payment Coordination Reform. The survey aimed to assess the effect of the reform to provide a decision-making basis for ensuring the “gatekeeper” role of the family doctor.

**Methods:**

A cross-sectional survey was conducted using a self-designed questionnaire in Changning District of Shanghai during January and February in 2014. Multi-stage random cluster was applied and 3040 residents were selected. Comparisons were made with statistically test between the contracted and non-contracted residents in four policy targeted dimensions, doctor-visiting behavior, health management and status, medical cost control and satisfaction.

**Results:**

Compared with the non-contracted residents, the contracted residents (72.9%) presented a higher prevalence rate of chronic diseases (32.6%), a higher proportion (51.9%) in first-contact in the community health service center and a higher proportion to refer to specialists as well (*P* < 0.001).The result showed that the average annual medical expense were significantly higher than non-contracted residents (*P* < 0.001), however, the difference disappeared after age, medical insurance and other socio-demographic variables were controlled. In terms of self-management of non-communicable diseases and complication prevention, the blood pressure control rate and blood glucose control rate for the contracted group were also higher than the counterparts, reaching up to 85.6 and 72.7% respectively.

**Conclusion:**

The preliminary analysis indicated that the contracted residents performed better in orderly doctor visiting behavior, health management behavior, health status and satisfaction. Follow up survey is necessary to further analyze the policy effect.

**Electronic supplementary material:**

The online version of this article (10.1186/s12875-019-0949-0) contains supplementary material, which is available to authorized users.

## Background

Family doctor is taken as the main carrier of primary health care (PHC) which provides comprehensive, continuous and appropriate health management [1]. According to current studies, the health problems of more than 90% of the population can be addressed by family doctor [2]. At present, the family doctor service system is carried out in over 50 countries and regions world widely, including Britain, Canada, the United States, Australia, Cuba and etc. First-contact in community health service centers (CHSCs) and referral via CHSCs implemented by family doctors have played as the active role of the “gatekeeper” of health systems, expecting to efficiently take advantage of health resources, control medical cost and improve health status [3, 4]. It was reported that PHC services and community health education provided by family doctors could relieve up to 70% of disease burden [5]. With the establishment of the long-term and stable partnership between family doctors and community residents, it is more likely to detect and assess the health risks for contracted residents, to restrain the incidence of non-communicable diseases (NCDs) and complications [6, 7], to reduce hospitalization probability [8–10] and overdose as well [11]. Additionally, the comparative studies of high-income countries have showed that the higher the proportion of family doctors, the lower the country’s overall health cost and the higher the quality evaluation score [12, 13].

In 2009, China launched a new round of health system reform, in which one of the five major targets was to establish a sound basic medical and health service system, and the key measurement was to develop and strengthen CHSCs to achieve universal health coverage. In recent years, the capability of CHSC has been significantly improved in an attempt to set up profit incentive mechanism; however, the primary health care system was less attractive to residents, thus the hierarchical diagnosis and treatment system had not been established as expected [14]. With the official issuance of *The Guidance for the General Practice System Establishment* [15] by the Chinese government in 2011 and based on the original service mode of the community Family Doctor team, several well-developed cities as Beijing, Shanghai, Shenzhen began to establish the family doctor system by facilitating the local residents to sign with family doctors, and to utilize CHSC-based first contact and referral services by appointment. Nevertheless, compared with the well-developed regions overseas, the system still has much to do. As indicated by the published literature, on one hand, the system of family doctor needs to improve service package design, collaborative referral mechanism, personnel training and etc. And on the other hand, the major problem the family doctors confronting with was absence of passion and enthusiasm [16, 17].

To deal with the less attractive (to residents) and absence of enthusiasm (to family doctors), Changning District of Shanghai, one of the first pilot areas in Shanghai and also in China practicing family doctor system, initiated Family Doctor and Medical Insurance Payment Coordination Reform in 2013 [18–20]. Two main measurements were implemented to promote family doctor system by cooperating with medical insurance institutions [21]. One measurement was establishment of standard Family Doctor Contracted Service Package with 36 service items. Based on demand survey, the package was designed to improve residents’ willingness to sign with family doctors, with all services covered in urban medical insurance scheme including Urban Employee Basic Medical Insurance and Urban Resident Basic Medical Insurance, and universal coverage of social medical insurance in urban China was the reform premier [22]. After signing with a family doctor, residents could select to utilize any CHSC package services, including free physical examination, personal health profile establishment, personal health intervention plan, NCD management services, referral to specialist without waiting in queue et al. On the other hand, this reform firstly connected family doctor contracted services to primary health workers’ income. Restoring Budgetary Control policy, or called “separation between revenue and expenditure” policy, was applied in CHSC in China since 2006 to avoid profit pursuit behavior of basic health care institution, however, which also reduced working enthusiasm for primary health workers. Thus, this reform established a motivation mechanism for family doctors to attract residents to sign with them and provide contracted services actively to keep a stable contraction relationship by contraction service fee (10 Yuan/person/month) paid directly by urban medical insurance institute. An evaluation system was also established to ensure the contraction was effective before payment, including CHSC visit rate, family doctor visit rate, referral (to specialists) rate, NCD management rate et al. 2500 residents was thought to be a reasonable contraction number of people for a family doctor to manage, supported by assistant nurses. 20,000 Yuan could be obtained additionally each month except for basic salary (if the effective contraction rate was set to be 80%). In this reform, cooperated between health care department and health insurance department, aiming to fulfil universal coverage of family doctor system, to implement health management through contracted services, to avoid medical expense waste in the case of disordered doctor visiting (i.e. visit specialists directly even with very common diseases) and to keep a stable doctor-patient relationship between family doctors and residents.

This reform via powerful leverage was expected to promote general practice system, to change the supply pattern of medical services, and to improve the residents’ doctor visiting behavior. Thus the medical resources can be shifted to communities and grassroots, and the policy targets, including improving the effect of disease management, controlling medical costs and enhancing overall patient satisfaction can be achieved [23]. However, no literature has analyzed the effect of such policy targets; thus it still remains unknown. The current study aimed to make a comparison between the contracted and non-contracted residents and make a comprehensive analysis of the Family Doctor and Medical Insurance Payment Coordination Reform in four dimensions: doctor-visiting behavior, health management effect, medical expense control and specific satisfaction of CHSC, which could provide evidences and suggestions in promoting the gate-keeper role of family doctors.

## Methods

### Sampling

We conducted a questionnaire survey in Changning District (See Additional file 1), which was located in the central and western part of Shanghai and covering ten communities/towns. The study population was composed of the permanent residents aged 15 and above who had been living there for at least 6 months. To ensure the demographic representation, the sample quantity was calculated using the formula below with the sample size *n* = 2780 [24, 25]. Specifically, confidence level α = 0.05 (two-sides), signing rate π = 0.22 (estimated according to the report data of the Administrative Departments of Public Health), relative error value *r* = 7%, allowable error δ = 7% × 22% = 0.0154. With no response rate controlled within 5%, the actual sample size was 2919 residents, i.e. the sample size in average for each street/town was about 300.

$$ n=\frac{\mu_a^2\pi \left(1-\pi \right)}{\delta^2} $$

The multi-stage random cluster sampling was adopted to obtain the selected individuals. In the first stage, we selected four neighborhood committees from each sub-street by probability-proportional-to-size sampling (PPS sampling) [25]; In the second stage, two communities were extracted from each neighborhood committee using simple random sampling; In the third stage, 38 households were selected from each community; In the last stage, we picked up one resident aged at least 15 years old as the ultimate participant via random drawing method of KISH table [25–28].

### Measures

Focus on the research purpose and based on current studies, we drafted *Questionnaire of Family Doctor Contracted Service and Overall Patient Satisfaction Assessment* in Changning District. Subsequently, two waves of expert consultation was conducted to revise the questionnaire structure and specific items, including academic researchers and family doctor team members from CHSCs. In addition, 15 residents were selected from Jiangsu sub-street Community of Changning District for a pre-site survey to revise the items which were difficult to understand or to answer. Based on the reform target, we designed our questionnaire, which was mainly composed of four parts: demographic characteristics in sociology, utilization of health care and costs, health management of chronic disease and satisfaction.

- **Characteristics of population sociology**: gender, age, education level, marital status, occupation, registered permanent residence, chronic disease history, contract signing and etc.
- **Health care utilization and costs**: the most recent illness type and treatment option, the utilized organization for health care, the yearly spending of personal health care, the total cost of the current medical treatment and individual payment, and other items such as referral experience, referral service mode, responsivity and etc.
- **Chronic disease management:** self-management of high blood pressure or diabetes or other chronic diseases, community involvement, chronic complication prevention, blood pressure and blood glucose monitoring, hypertension or diabetes control and etc.
- **Overall patient satisfaction with CHSC:** waiting time, diagnosis and treatment duration, medical equipment, technological level, service attitude, charged rate and medical environment and etc.

### Data collection

During January to February 2014, a household survey was conducted in Changning District of Shanghai. the investigators were recruited from Fudan University and Pudong Institute of Health Development Research. Beforehand, they were trained to better understand the survey questionnaire and were informed of household survey instruction. A small gift was sent to each respondents to improve the responding rate. In case of the household absence, the selected household could be replaced by the nearby neighborhood, but the replacement rate was strictly controlled within 5%.

The investigators checked the questionnaire after the respondent finished the questionnaire, including missing, wrong filling, et al. Moreover, the questionnaires were required to be rechecked after collected. 10% of the questionnaires were spot-checked via telephone interviews so as to ensure the quality and effectiveness of the survey. Epidata3.1 software was adopted to establish the dataset, and double parallel entry rule was applied in inputting data.

### Statistical analysis

The data analysis was performed with SPSS for Windows Version 18.0, the threshold of statistical significance set at *P* < 0.05 (two-tailed). The status of the health service use and cost, the effect of chronic disease management and the satisfaction with CHSCs were described using the statistical indicators such as median (Median), interquartile range (IQR), constituent ratio (%), etc. The simple differences between the contracted and non-contracted residents in four aspects of doctor visiting preference, health management, health expense and specific satisfaction were analyzed using Chi-squared test (*χ*^*2*^ tests). The medical spending was showed to be a severe right-skewed distribution by K-S test, thus we studied the impact of contraction on medical expense by Mann-Whitney nonparametric test.

### Ethics approval

The current survey was approved by the ethics committee of Pudong Institute for Health Development, Shanghai (No: PDWSL2013–1) which was in compliance with the Helsinki Declaration of 1964. All participants were voluntary and our investigators obtained selected residents’ consent to participate in before investigation, which guaranteed the verification and validation of the survey data at the same time. The questionnaires were anonymous, and the data obtained were only used for research purposes.

## Results

### Demographic and socioeconomic characteristics

Two thousand nine hundred forty-five valid questionnaires were collected, in which 2886 were valid, with the efficiency rate of 98.0%. Sample characteristics were described in Table 1. Specifically, 621 signed with one family doctor, with a signing rate of 21.52%, and 1192 (41.3%) suffered from at least one NCD, most of which were high blood pressure and diabetes. The female participants accounted for 59.9%; those aged 60 or above was 44%; the married accounted for 73.3%; High school or below accounted for, 70%. 1103 residents (38.2%) were in the state of employment; 360 (12.6%) were migrants. The comparison of the contracted and the non-contracted residents indicated that the two groups were statistically significant different (*P* < 0.05) in population sociology, except for gender. The NCD rate was significantly higher in the contracted than the counterpart.

**Table 1:** Socio-demographic characteristics of the sample group participated in this study

Characteristics	Category	N (%)		
		ALL (*N* = 2886)	Non-contacted (*N* = 2265)	Contacted (*N* = 621)^a^
Sex	Male	1158 (40.1)	922 (40.7)	236 (38.0)
	Female	1728 (59.9)	1343 (59.3)	385 (62.0)
Age (years)	≤44	801 (27.8)	741 (32.7)	60 (9.7)**
	45–59	815 (28.2)	671 (29.6)	144 (23.2)
	≥60	1270 (44.0)	853 (37.7)	417 (67.1)
Marital status	Single	770 (26.7)	628 (27.7)	142 (22.9)*
	Married	2116 (73.3)	1637 (72.3)	479 (77.1)
Education	Middle school or below	1024 (35.5)	745 (32.9)	279 (44.9)**
	High school	952 (33.0)	744 (32.8)	208 (33.5)
	College or equivalent	871 (30.2)	739 (32.6)	132 (21.3)
	Graduate degree and above	39 (1.4)	37 (1.6)	2 (0.3)
Employment	Employed	1103 (38.2)	948 (41.9)	155 (25.0)**
	Unemployed	1783 (61.8)	1317 (58.1)	466 (75.0)
Migrant status	Non-migrant	2526 (87.5)	1946 (85.9)	580 (93.4)**
	Migrant from other District	204 (7.1)	170 (7.5)	34 (5.5)
	Migrant from other city	156 (5.4)	149 (6.6)	7 (1.1)
Chronic disease^b^	Present	1192 (41.3)	739 (32.6)	453 (72.9)**
	Absent	1694 (58.7)	1526 (67.4)	168 (27.1)

* *P* < 0.05; ** *P* < 0.001

a. The indicated *P*-values from Pearson *χ*^*2*^ tests in which the socio-demographic characteristics recorded were compared between non-contacted and contacted participants

b. Hypertension, diabetes, chronic respiratory disease, cerebral apoplexy, chronic cardiovascular disease, gastrointestinal disorders, tumor and arthritis

### Doctor visiting behavior

As depicted in Table 2, the percentage of visiting doctors if sick lastly for the contracted residents was significantly higher than the non-contracted ones (*P* < 0.001), and the preference of medical institute was also significantly different (*P* < 0.001). In terms of the common diseases such as respiratory infection, the contracted residents preferred first-contact in CHSC (77.6%), but disease categories showed no impact on medical institution preference. Besides, an overall rate for referral behavior was 3.4% for the non-contracted, while the referral rate for contracted residents was significantly higher (*P* < 0.001) (Table 2). Besides, the contracted residents were more likely to refer to specialists via family doctors (65.9%), while that percentage for the non-contracted residents was only 39% (*P* < 0.001).

**Table 2:** Participants’ choice for medical services utilization in Changning district

	Category	N (%)		
		ALL	Non-contacted	Contacted^a^
Visit for a recent illness^b^	Yes	1509 (61.9)	1077 (57.0)	432 (78.8)**
	No	927 (38.1)	811 (43.0)	116 (21.2)
Visit for URI	Yes	355 (33.5)	288 (31.3)	67 (47.9)**
	No	706 (66.5)	633 (68.7)	73 (52.1)
Visit for NURI	Yes	1154 (83.9)	789 (81.6)	365 (89.5)**
	No	221 (16.1)	178 (18.4)	43 (10.5)
Medical institution of visit for a recent illness^b^	TCH	390 (25.8)	293 (27.2)	97 (22.5)**
	SCH	548 (36.4)	437 (40.6)	111 (25.6)
	CHSC	571 (37.8)	347 (32.2)	224 (51.9)
Medical institution of visit for URI	TCH	45 (12.7)	41 (14.2)	4 (6.0)**
	SCH	147 (41.4)	136 (47.3)	11 (16.4)
	CHSC	163 (45.9)	111 (38.5)	52 (77.6)
Medical institution of visit for NURI	TCH	345 (29.9)	252 (31.9)	93 (25.5)**
	SCH	401 (34.7)	301 (38.1)	100 (27.4)
	CHSC	408 (35.4)	236 (30.0)	172 (47.1)
Referral experience	Yes	82 (3.4)	38 (2.0)	44 (8.0)**
	No	2354 (96.6)	1850 (98.0)	504 (92.0)
Referral method	FD	34 (41.5)	5 (13.2)	29 (65.9)**
	NFD	35 (42.6)	27 (71.0)	8 (18.2)
	Both	13 (15.9)	6 (15.8)	7 (15.9)

**P < 0.001

a. The indicated P-values from Pearson χ2 tests in which the recorded choices for medical services utilization were compared between non-contacted and contacted participants

b. A latest illness that occurred within the six months before the survey

### Community health management for chronic diseases

The current survey showed that 57% of the residents with high blood pressure or diabetes had implemented self-management. The contracted residents had a higher percentage of self-management than the non-contracted (74.3% vs. 45.6%, *P* < 0.001). We found that 59.3% of the respondents with hypertension or diabetes were involved in the prevention and treatment of chronic disease complications. The proportion of the contracted patients (83%) was significantly higher than that of the non-contracted ones (43.9%) (*P* < 0.001). Table 3 showed that the blood pressure monitoring of the contracted patients was significantly better than that of the non-contracted patients (*P* < 0.001), while there was no significant difference in regular monitoring for blood glucose (*P* > 0.05). Hypertension or diabetes patients were more likely to measure blood pressure or blood glucose daily or weekly than non-contracted groups. The stable blood pressure control rate for the contracted residents was 85.6%, which was significantly higher than that of the non-signed patients (*P* < 0.01). The stable blood glucose control rate for the contracted patients was 72.2%, however, which was not statistically different from the non-contracted patients (*P* > 0.05).

**Table 3:** Community health management for participants’ chronic diseases in Changning District

	Category	N (%)		
		ALL	Non-contacted	Contacted^a^
NCD self-management	Carried out	564 (57.0)	272 (45.6)	292 (74.3)***
	Not carried out	425 (43.0)	324 (54.4)	101 (25.7)
Prevention of hypertension / diabetes complications	Carried out	575 (59.3)	258 (43.9)	317 (83.0)***
	Not carried out	395 (40.7)	330 (56.1)	65 (17.0)
Monitoring of hypertension	At least 1 times a day	159 (17.5)	86 (16.1)	73 (19.6)***
	At least 1 times a week	331 (36.5)	174 (32.5)	157 (42.1)
	At least 1 times a month	220 (24.2)	133 (24.9)	87 (23.3)
	Less than 1 times a month	21 (2.3)	16 (3.0)	5 (1.3)
	Unfixed	177 (19.5)	126 (23.6)	51 (13.7)
Diabetes surveillance	At least 1 times a day	22 (4.7)	14 (5.1)	8 (4.1)
	At least 1 times a week	73 (15.6)	44 (16.0)	29 (14.9)
	At least 1 times a month	98 (20.9)	55 (20.0)	43 (22.2)
	Less than 1 times a month	59 (12.6)	30 (10.9)	29 (14.9)
	Unfixed	217 (46.3)	132 (48.0)	85 (43.8)
Control of hypertension	Very stable	703 (81.0)	393 (77.7)	310 (85.6)**
	Generalized	122 (14.0)	87 (17.2)	35 (9.7)
	Unstable	43 (5.0)	26 (5.1)	17 (4.7)
Control of Diabetes	Very stable	254 (68.5)	145 (65.9)	109 (72.2)
	Generalized	80 (21.6)	52 (23.6)	28 (18.5)
	Unstable	37 (10.0)	23 (10.5)	14 (9.3)

* *P* < 0.05; ** *P* < 0.01; ***P < 0.001

^a^The indicated P-values from Pearson χ2 tests in which the recorded community health managements for chronic diseases were compared between non-contacted and contacted participants

### Personal health spending for medical care

The median of the medical expense was 1000 Yuan; The contracted residents were found to spend significantly more than the non-contracted (*P* < 0.001). However, there was no difference between two group in the medical expense for the latest medical treatment (*P* > 0.05). Table 4 showed that there was no significant difference in medical expense between contracted and non-contracted residents except for those who were over 60 years old and without NCDs (*P* > 0.05). In terms of those who suffered from upper respiratory disease (URD), the expense of the contracted residents for the latest medical treatment was significantly lower than that of the non-contracted (*P* < 0.05). When the medical institution variable was considered, there was no significant difference in the medical expense for the latest medical treatment between the contracted and non-contracted residents in respiratory diseases (*P* > 0.05).

**Table 4:** Personal health spending for participants’ medical care in Changning district

	Category	Median(IQR)		
		ALL	Non-contacted	Contacted ^a^
personal health spending/year		1000 (1500)	1000 (1300)	1500 (1600) **
NCD* Age(years)	Present * ≤44	1800 (1400)	1200 (1600)	2000 (1500)
	Present *45–59	1500 (1850)	1500 (2000)	1500 (1000)
	Present * ≥60	2000 (2000)	1800 (2000)	2000 (2000)
	Absent * ≤44	300 (410)	300 (355)	200 (900)
	Absent * 45–59	500 (800)	500 (800)	800 (700)
	Absent * ≥60	700 (700)	600 (600)	1000 (1500) **
personal health spending for last visit		30 (195)	30 (196)	22 (190)
Disease	URI	20 (50)	20 (60)	12.5 (41) *
	NURI	40 (240)	50 (193)	30 (290)
Disease*Medical institution of visit	URI * tier-3	20 (94)	20 (94)	60 (213.5)
	URI * tier-2	30 (90)	30 (90)	20 (70)
	URI * CHSC	10 (30)	6 (20)	10 (30)
	NURI * tier-3	200 (970)	200 (870)	300 (2212)
	NURI * tier-2	100 (285)	91.5 (238.75)	195 (580) **
	NURI *CHSC	10 (30)	10 (25)	13 (30)

* *P* < 0.05; **P < 0.001

a. The indicated P-values from Mann-Whitney U tests in which the recorded out-of-Pocket payments for medical care were compared between non-contacted and contacted participants

### Satisfaction

As indicated by Table 5, the satisfaction evaluation of CHSC’s services showed that residents were more satisfied (including “very satisfied” and “satisfied”) in service attitude (93.8%), charged price (93.4%) and convenient degree (93.2%), and were less satisfied in medical equipment (72.7%), waiting time (76.1%) and diagnosis and treat duration (82.6%). In general, the percentage of satisfied (including very satisfied and satisfied) in contracted residents were significantly higher in convenient degree, waiting time, diagnosis and treat duration, medical environment, medical equipment and technical skill, service attitude and charged price. In particular, the dissatisfaction proportion of the diagnosis and treat duration was up to 20%, which could be explained by the fact that the non-contracted residents rarely utilize appointment service in CHSCs.

**Table 5:** Participants’ satisfaction evaluation of community health services in Changning district

	Category	N(%)		
		ALL	Non-contacted	Contacted^a^
Convenient degree	Very satisfied	915 (62.7)	528 (59.1)	387 (68.3)**
	Comparatively satisfied	446 (30.5)	301 (33.7)	145 (25.6)
	Dissatisfied	99 (6.8)	64 (7.2)	35 (6.2)
Waiting time	Very satisfied	451 (32.2)	241 (28.4)	210 (38.2)**
	Comparatively satisfied	614 (43.9)	384 (45.2)	230 (41.8)
	Dissatisfied	335 (23.9)	225 (26.5)	110 (20)
diagnosis and treat duration	Very satisfied	522 (37.8)	278 (33.2)	244 (44.9)**
	Comparatively satisfied	619 (44.8)	392 (46.8)	227 (41.8)
	Dissatisfied	240 (17.4)	168 (20.0)	72 (13.3)
Medical environment	Very satisfied	634 (44.7)	348 (40.2)	286 (51.7)**
	Comparatively satisfied	615 (43.4)	401 (46.4)	214 (38.7)
	Dissatisfied	169 (11.9)	116 (13.4)	53 (9.6)
Medical equipment	Very satisfied	444 (33.1)	250 (30.6)	194 (37.0)*
	Comparatively satisfied	531 (39.6)	329 (40.3)	202 (38.5)
	Dissatisfied	366 (27.3)	237 (29.0)	129 (24.6)
Technical skill	Very satisfied	804 (55.0)	460 (51.4)	344 (60.7)**
	Comparatively satisfied	514 (35.2)	334 (37.3)	180 (31.7)
	Dissatisfied	144 (9.8)	101 (11.3)	43 (7.6)
Service attitude	Very satisfied	819 (56.9)	440 (50.1)	379 (67.4)**
	Comparatively satisfied	531 (36.9)	372 (42.4)	159 (28.3)
	Dissatisfied	90 (6.3)	66 (7.5)	24 (4.3)
Charged price	Very satisfied	1000 (68.5)	582 (65.2)	418 (73.7)**
	Comparatively satisfied	364 (24.9)	244 (27.3)	120 (21.2)
	Dissatisfied	96 (6.6)	67 (7.5)	29 (5.1)

* *P* < 0.05; **P < 0.001

a. The indicated P-values from Pearson χ2 tests in which the recorded satisfaction of community health services were compared between non-contacted and contacted participants

## Discussion

In the current survey, the sociodemographic characteristics of the sample were consistent with the officially issued demographic feature of population in Changning District, except for a relatively lower proportion in people aged below 35 and higher proportion older than 60. According to the literature, the application of Kish table could bring about lower responding rate of young respondents, resulting in gender and age structure distortion [24]. Besides, the signing rate was found to be 21.52% according to this study, which was slightly lower than the one published by the Community Health Management Center of Changning District (25.18%) [29]. Generally, the sample was representative to carry out the study.

Changning was the first to implement the Family Doctor and Medical Insurance Payment Coordination Reform, aiming to facilitate family doctors to provide PHC. The major problem of the reform is how to attract the local residents to sign with the family doctors. Table 1 showed that the current residents contracted with family doctors in Changning District were mainly the elderly or NCD patients. The local government usually gave priority to the poor, NCD patients, the elderly and the disabled, who had higher demands and level of utilization for community health services. At the same time, these groups of population were generally more supportive to implement family doctor policy, which was favorable to expand the coverage of contracted population in the initial stage of policy implementation [18, 30]. However, it could not be ignored that most of the contracted residents had high demands for health care services, so family doctors’ workload once kept increasing significantly. In addition, after family doctor contracted services were gradually recognized and accepted by key clients, service objects should be expanded to the whole population gradually. The government should design contract service content according to specific demands of different population in order to further promote the sustainable development of family doctor contract service system.

Another problem is how to encourage family doctors to provide substantial contract services meanwhile. The contracted service fee compensation mechanism implemented by the local government is able to improve family doctors’ income structure, and can establish the assessment mechanism to achieve the principle of “pay more for serve more” [21]. With the improvement of family doctors’ motivation level, our study showed (Table 2) that the medical service demands of contracted residents had been met better, whether among the common URD patients or other patients, and the residents’ utilization of services was improved as well. This improvement brought about better health status for contracted residents, which could be contributed to a stable and trustworthy relationship with family doctors [31], but also the referral patterns. In terms of referral preferences, we found that the contracted residents (51.9%) were more likely to choose CHSC as their first choice of seeking medical advice for common diseases such as cold and cough while the non-contracted residents tended to select tier-2 and tier-3 hospitals (67.8%). This suggested that the family doctor policy might have played the role of attracting residents to first-contact in CHSCs as their first choice and referring to specialists via CHSCs preliminarily, which had been expected by the policy designer [14].

In 2008, the World Health Organization emphasized that the continuity and comprehensiveness of CHSCs were significant determinants to effectively implement PHC services such as chronic disease management, disease prevention and control, elimination of health risk factors [10, 32, 33]. By strengthening the family doctor’s advantages of patient-centered services, chronic disease management, health monitoring, bridging the traditional boundary between diagnosis and treatment, PHC can effectively improve population’s health status [23]. The current survey (Table 3) indicated that under the guidance of family doctors, the proportions of self-management and complications’ prevention for NCDs among contracted residents were much higher than that of non-contracted residents. Compared with the blood pressure monitoring behavior (80.5%), the blood glucose monitoring behavior (53.7%) was worse in contracted diabetic residents, but still better than the non-contracted residents [34]. It is possible to develop the orderly doctor visiting behavior of first-contact in CHSCs by establishing a sustainable partnership between contracted residents and family doctors, to better manage the NCDs by monitoring and prevention of complications in community, to make full use of Knowledge & Attitude & Practice (KAP) Health Education Model, and to improve the disease control on high blood pressure and diabetes effectively [35].

The new medical reform in China targeted in “effectively relief residents’ economic burden in health care” as well. Personal health expenditure (OOP) referred to the expenses paid by residents themselves in the process of using medical and health services, which was the main indicator to measure the economic burden of residents’ medical care [35]. Theoretically, contracted services can conduct residents to orderly seek medical resources from the gatekeeper level, so that they are able to diagnose their medical problems and complications for early treatment, reducing the induced consumption from unnecessary advanced medical services [23]. However, the current survey data (Table 4) were not fully consistent with the theory. In terms of medical expense for per treatment/person, the contracted residents were lower than the non-contracted, which was affected by the preference of medical institution. Previous evidence also showed that the health-care cost could be much lower if the contracted residents were more likely to select CHSCs as their first choice for common diseases [34]. However, compared with the non-contracted individuals, the average annual medical expense of contracted residents did not show absolute advantage, even if considering the health status and age structure. On one hand, the average annual medical spending could be affected by treatment frequency, i.e. the majority of the contracted residents were elderly people who demand and utilize medical care relatively more. On the other hand, family doctor was initially established in Shanghai and initially coordinated with the medical insurance reform in 2013, thus the policy effect might not show up promptly within one year.

The level of satisfaction of community health services is an important metrics and means to improve residents’ compliance with family doctors and stabilize the relationship between them [23]. According to our survey, the proportion of residents who expressed “very satisfied” or “satisfied” for the evaluation of community health services was more than 70.0% (Table 5). Further comparison showed that Changning District of Shanghai has strengthened the overall ability of family doctors of treatment after ten-years’ exploration in primary health care, leading to relatively positive results of satisfaction and recognition, providing a good foundation for the contracted service payment reform [19]. In addition, the contracted residents are more likely to be “very satisfied” or “satisfied” about CHSC’s services than the non-contracted. The top two indicators of satisfaction items were waiting time (20%) and medical equipment (24.6%), which was basically the same as the non-contracted residents.

### Limitation

Firstly, this study is a preliminary analysis of the effect of the coordinated reform related to family doctor contracting services and medical insurance payment. Some effect, such as the control of medical expenses, the outcome of health management, the complication and morbidity, are not outstanding, which is probably because that the policy implementation period is relatively short (1 year) or the reform effect often has a certain lag. Therefore, the research team is following up the respondents to further conduct cohort study. Secondly, how to effectively eliminate the influence from other reform policies in the effect analysis to obtain a net effect is a common bottleneck confronted by social experimental research. The family doctor contracted service is the core of the medical reform in Changning District of Shanghai and even in China. The relevant policies implemented by different departments in recent years are coordinated closely, making the family doctor policy study more complicated. Thirdly, the medical expense involved in the study are mainly based on the subjective memories of the respondents rather than historical data from the medical information system, which could be bias to some degree. However, we tried to collect medical expense data by verifying the respondents’ medical bills and repeadly checking the answer to get more precisely answer.

## Conclusion

Shanghai, one of the earliest cities of practicing primary health care in China, initiated the Family Doctor and Medical Insurance Payment Coordination Reform. This is an initial study to preliminarily analyze the policy effect expected by the health care government. We found contracted residents performed better in doctor-visiting and health management behavior, and were better in health and satisfaction outcome. Follow-up survey is necessary to further analyze the policy effect of the two groups.

## Additional file

Additional file 1: Questionnaire of Family Doctor Contracted Service and Overall Patient Satisfaction Assessment. (DOCX 32 kb)

## Abbreviations

CHSC: Community health service center
NCD: Non-communicable diseases
NURI: Non-upper respiratory infection
PHC: Primary health care
URD: upper respiratory Disease

## References

[1] Taylor RB (1998). Family medicine: current issues and future practice family medicine-principles and practice.

[2] Dupuit S, Collins E, Shergill S (1998). Computer-based assistance in family medicine. Comput Methods Prog Biomed.

[3] Zhang Y, Li YL (2005). Characteristics and enlightenment of foreign GPs. Chin J Hosp Manag.

[4] Zhang Y, An M, Cai WQ (2011). The enlightenment of foreign family doctor system to the community health management of our country. J Community Med.

[5] Kripalani S, LeFevre F, Phillips CO (2007). Deficits in communication and information transfer between hospital-based and primary care physicians: implications for patient safety and continuity of care. Jama.

[6] Smetana GW, Landon BE, Bindman AB (2007). A comparison of outcomes resulting from generalist vs specialist care for a single discrete medical condition: a systematic review and methodologic critique. Arch Intern Med.

[7] Mendis S, Johnston SC, Fan W (2010). Cardiovascular risk management and its impact on hypertension control in primary care in low-resource settings: a cluster-randomized trial. Bull World Health Organ.

[8] Woodward CA, Abelson J, Tedford S (2004). What is important to continuity in home care?: perspectives of key stakeholders. Soc Sci Med.

[9] Weinberger M, Oddone EZ, Henderson WG (1996). Does increased access to primary care reduce hospital readmissions?. N Engl J Med.

[10] World Health Organization (2008). The world health report 2008: primary health care now more than ever.

[11] Pongsupap Y, Lerberghe WV (2006). Choosing between public and private or between hospital and primary care: responsiveness, patient-centeredness and prescribing patterns in outpatient consultations in Bangkok. Tropical Med Int Health.

[12] Baicker K, Chandra A. Medicare Spending, The physician workforce, and beneficiaries” quality of care. 2004. 10.1377/hlthaff.w4.184 15451981

[13] Rose JH, O'Toole EE, Dawson NV (2000). Generalists and oncologists show similar care practices and outcomes for hospitalized late-stage cancer patients. Med Care.

[14] Jiangnan C. Why cannot hospital health alliance be successfully implemented. China Soc Secur. 2011(9):82–3.

[15] State council. Directions on the establishment of the general practitioner system, July 2011.

[16] He XL, Liang H (2012). Theoretical discussion and policy proposal to promote the reform of family responsibility doctors system. Chin J Health Policy.

[17] Du LJ, Lin WL. Analysis of the problems and countermeasures existing in the policy insurance of community family doctor system. Chinese Rural Health Service Administration2, 013, 33 (8): 876–878.

[18] Ge M, Jiang P, Lu W (2012). The discussion about pushing approach, service mode and system architecture of family doctor system: taking Changning as an example. Chin Health Resour.

[19] Changning District Health Bureau of Shanghai (2013). Mode and mechanism innovation of family doctor system - Shanghai Changning experiment.

[20] The NDRC, Startup of the pilot reform of GP practice and service mode [EB / OL].http: // www sdpc.gov.cn / shfz/ yywstzgg / ygdt / t20120830_502402.htm, 2012-08-28.

[21] Wu J, Shi Q. Consideration on the reform of family doctor contracted service and the mode of medical insurance payment. Chin Gene Pract. 2008;(5):34–6.

[22] Meng Q, Fang H, Liu X, Yuan B, Xu J (2015). Consolidating the social health insurance schemes in China: towards an equitable and efficient health system. Lancet.

[23] Lu W, Zhang YM, Liang H (2016). Analysis of the theoretical basis and policy value for family doctor contracted service and the co-reform of medical insurance payment. Chin J Health Policy.

[24] Babbie E (2009). The Practice of Social Research.

[25] Feng XT (2001). Research methods of sociology.

[26] Zhan SK (2003). Field investigation technology.

[27] Cao Y, Chen J, Cao JW (2004). Kish grid sampling applied inworldwide health survey(China survey). J Fudan University: Med Ed.

[28] Zhang LP (2009). Home-entry sampling by use of Kish table-study of the age structure distortion of respondents. Sociol Res.

[29] Changning District Health Bureauof Shanghai (2014). Annual report of community health management center.

[30] Zhao Y, Zhu Y, Pan YH (2012). Study of the willingness and effect factors on the family doctors contracted with community outpatients. Chin Gen Pract.

[31] Liang WN, Lü ZF. General medical theory and practice. Beijing: People's medical publishing house; 2012.

[32] Shi L, Macinko J, Starfield B (2003). The relationship between primary care, income inequality, and mortality in US states, 1980–1995. J Am Board Fam Pract.

[33] Franks P, Fiscella K (1998). Primary care physicians and specialists as personal physicians. Health care expenditures and mortality experience. J fam Pract.

[34] Lu W, Zhang YM, Liang H (2016). Evaluation of implementation effect based on demander’s family doctor contracted service-focusing on chronic diseases. Chin J Health Policy Res.

[35] Liao Yn LY, Zhang C (2010). Analysis of KAP health education effect of the nutrition in Chinese and Western medicine for hypertension of the elderly in community. Chin J Gerontol.

