# Supplementary material for: A preliminary effect analysis of family doctor and medical insurance payment coordination reform in Changning District of Shanghai, China
Source: BMC Fam Pract. 2019 May 10;20:60. doi: 10.1186/s12875-019-0949-0 (PMC6511160; doi:10.1186/s12875-019-0949-0)
Supplement: Supplementary file 1 — Questionnaire of Family Doctor Contracted Service and Overall Patient Satisfaction Assessment. (DOCX 32 kb) [file 12875_2019_949_MOESM1_ESM.docx]

| **ID:** |
| --- |

**Questionnaire of Family Doctor Contracted Service and Overall Patient Satisfaction Assessment**

**Introduction for investigators**: We conduct this survey to evaluate family doctor contract service, to further provide contract services which required by the residents and provide appropriate suggestions to the government.

**About Confidentiality:** You are free to accept or refuse this survey. Once you accepted, your answers will be kept safely and just used for academic use. The questionnaire is anonymous.

| **Is it the selected residents?**  **Yes, input the selected address:**  Sub-district__________, Neighborhood Committee__________, Building___________, Room___________.  **No, input the replaced address:**  Sub-district__________, Neighborhood Committee__________, Building___________, Room___________.  **Investigator ID:** ____________, **Signature:** _________________  **Visit date:** ________Year, _________Month, _________Date  **Quality of this questionnaire: Good Fair Bad**  **Check Operator ID:** _____________, **Signature:** ______________ |
| --- |

**Part A. Demographic Information**

**A1** your birth date

_______ Year, _______Month, _______Date

**A2** your gender:

①female ②male

**A3** your nationality:

①Han ②Zhuang ③Manchu ④Hui ⑤Miao ⑥Uighur ⑦Yi ⑧Tujia ⑨Mongolian ⑩Korean ⑪Tibetan ⑫others

**A4** your education level:

①never received ②primary school graduated ③primary school graduation

④middle school graduation ⑤high school/secondary school/technical school

⑥college graduation ⑦undergraduate graduate ⑧master graduate, or higher

**A5** marital status:

①never married ②married ③cohabitation ④widowed ⑤divorced ⑥separated

**A6** medical insurance you are participating in **(multiple choice):**

①Urban workers basic medical insurance ②Public medical care

③Urban residents medical insurance ④New rural cooperative medical care ⑤Commercial medical insurance ⑥others ⑦no participate ⑧not clear

**A7** hukou or household registration location:

①Shanghai ②others

**A8** Have you retired yet?

①Yes ②No

**Part B. Cognitive and Contract Behavior**

**B1** Do you know or have heard of the family doctor?

①yes ②no

**B2** Do you know about family doctor contract services?

①yes ②no

**B3** Do you know the specific content of the family doctor service? **(multiple choice)**①not clear ②home treatment ③rehabilitation guidance ④family bed or care

⑤chronic disease or infectious disease visit ⑥elderly health care ⑦others

**B4** Did you sign with a family doctor?

①yes ②no

**B5** If you have not signed up yet, are you willing to accept the contract service?

①yes ②no ③not decided

**B6** Does any of your family member has signed with a family doctor?

①yes ②no

**Part C. Health and Service Use**

**C1** Last time you were sick, what disease is it?

(Disease name)

**C2** Did you visit a medical institution for treatment this time?

①yes ②no

**C3** Which level of medical institution did you visit for this illness?

①Tertiary hospital ② Secondary hospital ③Community Health Center

**C4** If you visit the second or third level hospital, what is the reason? **(multiple choice)**

①feel the condition is serious ②advanced medical technology

③there are familiar specialist in the hospital ④others

**C5** If you visit the community health center, what is the reason? **(multiple choice)**

①feel the condition is lighter ②convenient for treatment ③cheaper

④good health care attitude ⑤others

**C6** How much did you spent for doctor visiting this time ____________Yuan

**C7** Have you been referred to secondary and tertiary hospitals through the community health service center?

①yes ②no

**C8** If yes, the referral method: ①Family Doctor ②Community Health Center ③Both

**C9** Do you suffer from any chronic diseases?

①yes ②no

If yes, please answer C9-C

**C10** If you have a chronic disease, which of the following is it? How many years?

| Type | Suffering from the disease, check“√” | Illness years | Type | Suffering from the disease, check“√” | Illness years |
| --- | --- | --- | --- | --- | --- |
| hypertension |  |  | heart disease |  |  |
| diabetes |  |  | digestive disease |  |  |
| lung disease |  |  | tumor |  |  |
| stroke |  |  | arthritis |  |  |

**C11** What level of medical institutions do you usually visit for chronic diseases treatment?

①Community Health Center ②Secondary hospital ③Tertiary hospital

**C12** Will you visit a family doctor when you are sick?

①yes ②no ③depend on the situation

**C13** Under guidance of Family Doctor, have you initiated self-management?

①yes ②no

**C14** Have you received prevention service of hypertension / diabetes complications?

①yes ②no

**C15** Please evaluate the control effect of hypertension?

①Very stable ②Generalized ③Unstable

**C16** Please evaluate the control effect of diabetes?

①Very stable ②Generalized ③Unstable

**C17** If you are a hypertension patient, what is your monitoring frequency of hypertension?

①At least 1 times a day ②At least 1 times a week ③At least 1 times a month ④Less than 1 times a month ⑤Unfixed

**C18** If you are a diabetes patient, what is your monitoring frequency of diabetes?

①At least 1 times a day ②At least 1 times a week ③At least 1 times a month ④Less than 1 times a month ⑤Unfixed

**C19** How much did you spent in doctor visiting last year? ___________Yuan

**Part D. Satisfaction**

**D1** Are you satisfied with the convenience of CHSC services?

①Dissatisfied ②Comparatively satisfied ③very satisfied

**D2** Are you satisfied with the duration of waiting in line?

①Dissatisfied ②Comparatively satisfied ③very satisfied

**D3** Are you satisfied with diagnosis and treatment duration?

①Dissatisfied ②Comparatively satisfied ③very satisfied

**D4** Are you satisfied with the comfort level of the medical environment?

①Dissatisfied ②Comparatively satisfied ③very satisfied

**D5** Are you satisfied with the medical equipment used?

①Dissatisfied ②Comparatively satisfied ③very satisfied

**D6** Are you satisfied with the FDs’ technical skill?

①Dissatisfied ②Comparatively satisfied ③very satisfied

**D7** Are you satisfied with service attitude?

①Dissatisfied ②Comparatively satisfied ③very satisfied

**D8** Are you satisfied with charged prize?

①Dissatisfied ②Comparatively satisfied ③very satisfied

**Thank you for your participation !**
